# Population‐level multiplexing: A promising strategy to manage the evolution of resistance against gene drives targeting a neutral locus

**DOI:** 10.1111/eva.12945

**Published:** 2020-03-25

**Authors:** Matthew P. Edgington, Tim Harvey‐Samuel, Luke Alphey

**Affiliations:** ^1^ The Pirbright Institute Woking UK

**Keywords:** CRISPR, gene drive, genetic engineering, multiplexing, population alteration, resistance

## Abstract

CRISPR‐based gene drives bias inheritance in their favour by inducing double‐stranded breaks (DSBs) at wild‐type homologous loci and using the drive transgene as a repair template—converting drive heterozygotes into homozygotes. Recent studies have shown that alternate end‐joining repair mechanisms produce cut‐resistant alleles that rapidly induce drive failure. Multiplexing—simultaneously targeting multiple sites at the wild‐type locus—is commonly assumed to overcome this issue since resistance would need to develop at all target sites for the system to fail. This may work for some population suppression drives targeting essential (e.g. viability or fertility) genes if careful design can ensure cut‐resistant alleles themselves have low fitness. However, here, models are used to demonstrate that this approach will be ineffective when targeting neutral loci. We then go on to compare the performance of four alternative population‐level multiplexing approaches with standard individual‐level multiplexing. Two of these approaches have mechanisms preventing them from becoming linked, thus avoiding multiple simultaneous DSBs and giving a large improvement. Releasing multiple unlinked drives gives a modest improvement, while releasing multiple drives that may become linked over time produces a decrease in performance under the conditions tested here. Based on performance and technical feasibility, we then take one approach forward for further investigation, demonstrating its robustness to different performance parameters and its potential for controlling very large target populations.

## 1 INTRODUCTION

A range of gene drive systems have been proposed that are predicted to spread novel genes to high frequency in a population even if they confer a fitness cost on individuals carrying them (Alphey, 2014; Champer, Buchman, & Akbari, 2016). Of these, the best studied and most powerful are the “homing drives”—first proposed using homing endonuclease genes (HEGs) (Burt, 2003) and later CRISPR technology (Esvelt, Smidler, Catteruccia, & Church, 2014). In the latter, a Cas9 endonuclease is directed by a guide RNA (gRNA) to induce a double‐stranded DNA break (DSB) at a specific sequence within a wild‐type locus homologous to the drive transgene. Ideally, the gene drive construct is used as a repair template for this DSB in a process known as homology‐directed repair (HDR), effectively copying the drive construct onto the homologous chromosome (Esvelt et al., 2014). This process is referred to as homing and, if it occurs efficiently in the germline, can allow the gene drive to spread rapidly to a high frequency in a population (Alphey & Bonsall, 2014; Deredec, Burt, & Godfray, 2008; Deredec, Godfray, & Burt, 2011; Unckless, Messer, Connallon, & Clark, 2015).

Double‐stranded breaks, however, are not always repaired by HDR. A variety of end‐joining repair mechanisms such as nonhomologous end joining (NHEJ) can also repair DSBs, in effect by ligating the broken ends together (Burt, 2014; Deredec et al., 2008, 2011; Unckless, Clark, & Messer, 2017). These processes have been shown to be error‐prone and frequently result in the creation of insertion/deletion mutations that modify the DNA sequence at the gRNA target site, thus making it resistant to recognition by Cas9 and precluding further cutting/homing (Hammond et al., 2017; Kistler, Vosshall, & Matthews, 2015; Ren et al., 2013). If these resistant alleles confer a fitness advantage relative to the gene drive transgene, they are likely to spread, leading to eventual elimination of the gene drive system and loss of control (Beaghton et al., 2017; Deredec et al., 2011; Noble, Adlam, Church, Esvelt, & Nowak, 2018; Noble, Olejarz, Esvelt, Church, & Nowak, 2017; Prowse et al., 2017; Unckless et al., 2017)—a consequence demonstrated in recent empirical studies (Champer et al., 2017; Hammond et al., 2017, 2018; KaramiNejadRanjbar et al., 2018). This tendency to rapidly induce their own resistance is regarded as perhaps the greatest technical hurdle in developing CRISPR‐based gene drives, which will remain robust over biologically relevant timescales and population sizes (Hammond et al., 2017; Reed, 2017).

**FIGURE 1:**
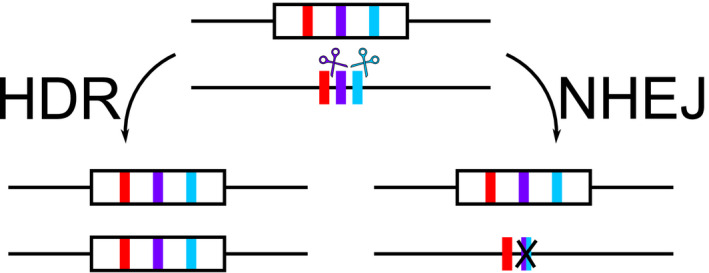
A schematic of the possible repair mechanisms when two DSBs are induced simultaneously by a multiplexed CRISPR gene drive system. Here, boxes represent transgenic constructs that contain multiplexed gRNAs (coloured bars) that target specific sequences on the target chromosome (coloured bars outside of transgenic constructs). In this example, DSBs are made at purple and cyan target sites. Within our model, there are two possible repair mechanisms (left) HDR and (right) NHEJ. For HDR, the whole multiplexed construct homes into the homologous chromosome and removes gRNA target sites due to the location of homology arms. In the case of NHEJ, the intervening sequence between the DSBs is deleted and the two ends ligated together resulting in resistance at these target sites (and all in between). Note that here NHEJ is used as a catch‐all term for the full range of possible end‐joining mechanisms

A widely cited strategy for overcoming resistance is to engineer gene drive transgenes to target multiple linked sites at the wild‐type locus through the simultaneous expression of several different gRNAs (Crisanti et al., 2016; Esvelt et al., 2014; Marshall, Buchman, Sánchez, & Akbari, 2017; Noble et al., 2017; Oberhofer, Ivy, & Hay, 2018; Prowse et al., 2017). This “multiplexing” of gRNAs is predicated on the theory that individuals would need to be/become simultaneously resistant at all gRNA target sites such that the drive is unable to target the homologous chromosome further—an event that decreases in likelihood with increasing numbers of gRNAs (Marshall et al., 2017). However, a rarely discussed but potentially significant drawback of currently proposed multiplexing designs is that simultaneous DSBs at multiple sites on the same chromosome can be repaired via NHEJ, resulting in the deletion of the entire intervening sequence (Brinkman et al., 2018; Champer et al., 2018; Kistler et al., 2015; Prowse et al., 2017; Ren et al., 2013). This would effectively create resistance at all target sites between those two (or more) DSBs, potentially in a single step (Figure 1). Where the goal of the gene drive design is to home into and consequently disrupt an essential gene (as for some suppression drives), these NHEJ deletion‐mutant alleles will be rapidly selected against as they are likely nonfunctional (Esvelt et al., 2014). However, when targeting a gene drive to a neutral locus (“a locus that has no effect on adaptation because all genotypes have the same fitness” (Allendorf, Hohenlohe, & Luikart, 2010)), this becomes a much more serious concern as drive‐resistant deletion mutants will likely retain full fitness, potentially allowing them to undergo positive selection (Prowse et al., 2017). With neutral drives spreading “cargo genes,” the basis of current efforts to spread malaria‐refractory genes in mosquitoes and suppressive sex‐ratio biases in invasive rodents, it is imperative that alternative robust resistance management strategies be developed.

Here, we introduce and demonstrate novel methods for the management of resistance to a gene drive targeting a neutral locus. These strategies differ fundamentally from the current paradigm in that they allow multiplexing to function at the population rather than individual level by integrating each gRNA onto an independently segregating construct. Firstly, we formulate a stochastic mathematical model of a “classic” multiplexed CRISPR gene drive system targeting *n* sites (validated in Figure S1). We show that where gRNAs are expressed simultaneously (current paradigm), such a system is likely to be ineffective at preventing transgene elimination when targeting a neutral locus but would drastically improve if gRNA action could be engineered to occur sequentially. The model is then adjusted for each population‐level multiplexing strategy—“separate,” “additive,” “overwriting” and “blocking”—with numerical simulation used to explore the predicted behaviour of each within a small population of 1,000 individuals. Results demonstrate that the separate, overwriting and blocking strategies each give an improvement over classical multiplexing for a neutral locus, whereas an additive strategy appears to be at a deficit to classical multiplexing. Finally, we conduct additional numerical simulations of the preferred “blocking” strategy to assess efficiency under a range of different performance parameters and biologically realistic population sizes.

## 2 RESULTS

### 2.1 Neutral‐locus multiplexing approaches modelled

In subsequent sections, we demonstrate the inability of classical multiplexing to effectively manage resistance. Then, we explore four alternative strategies that are outlined here. For ease of interpretation, each is described using a “global” design; that is, both Cas9 and the gRNA are located in one construct at a locus homologous to that targeted by the guide. However, the underlying logic still applies if either of these components was absent, for example, in the elements of a “daisy‐chain” system (Noble et al., 2019).

#### 2.1.1 Classic multiplexing

**FIGURE 2:**
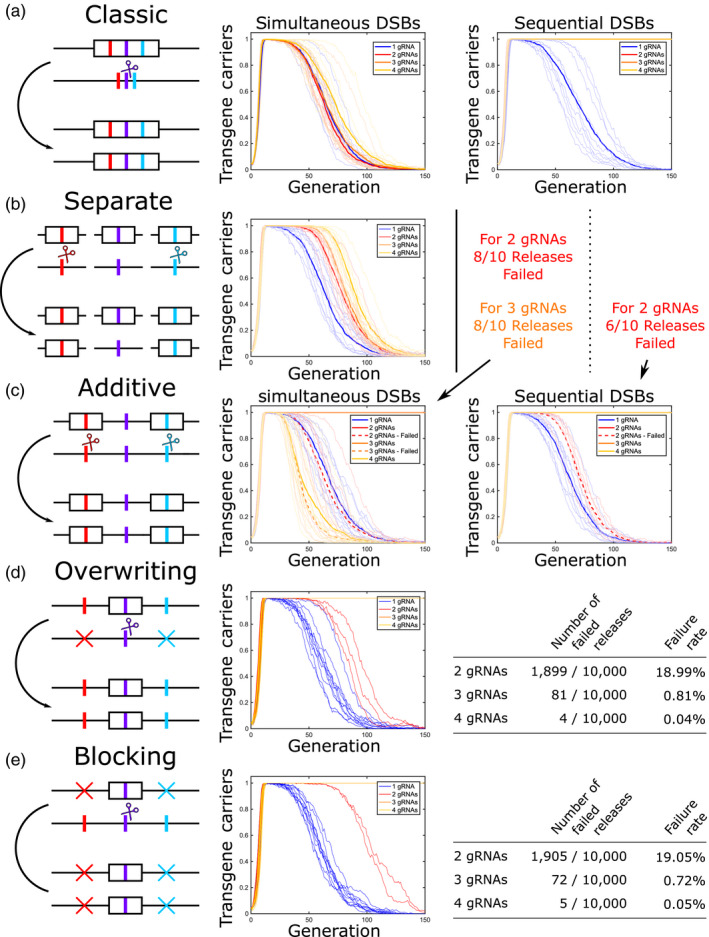
Schematic diagrams and simulated releases for each multiple target site “multiplexed” CRISPR gene drive approach modelled. Here, each row represents a multiplexing approach with (a) classical multiplexing, (b) separate unlinked gene drives, (c) additive approach, (d) overwriting approach and (e) blocking approach. Rows consist of a schematic demonstrating system function and numerical simulation results. In (a) and (c), DSBs may be simultaneous or sequential—indicated in headings. In all cases, thin lines represent single numerical simulation results, while thick lines show the mean over those simulations. Line colours/type details are given in legends. Panels (d) and (e) represent our most promising approaches and so additional numerical simulations were conducted. For visual clarity, we present the first ten numerical simulations from a set of 10,000 for each target site number. Results of all 10,000 simulations are summarized in the respective tables. In several panels, lines for successful introductions overlap each other—namely average lines for 2, 3 and 4 gRNAs in a—sequential DSBs; successful releases for 2 and 3 gRNAs in b—simultaneous DSBs; and successful releases for 2, 3 and 4 gRNAs in c—sequential DSBs. For panels (d) and (e), many successful releases overlap each other for 2, 3 and 4 gRNAs. All numerical simulations are conducted using release ratio 0.05 and a population of 1,000 individuals. Schematic diagrams further demonstrating expected interactions in the additive, overwriting and blocking strategies are given in Figures S5–S7

As a baseline against which to compare the proposed population‐level multiplexing designs, we first model a classic multiplexing strategy in which a single construct carrying multiple gRNAs targets several, tightly linked sites on the homologous chromosome (as in Figures 1 and 2(a)). Here, successful homing events result in the construct replacing the region spanning the complete set of target sites. We model two examples, namely (a) all guides are expressed and cut simultaneously and (b) each guide is expressed and cuts sequentially.

#### 2.1.2 Separate drives

Another approach that has been suggested in the literature is the release of multiple independent single‐target CRISPR gene drives similar to the idea discussed in Noble et al. (2017). Here, each distinct drive would behave exactly as expected for a single‐target CRISPR gene drive (see Figure 2(b)).

#### 2.1.3 Additive strategy

Rather than introducing a single construct targeting multiple linked sites, here we introduce a number of distinct constructs each targeting a single site, sufficiently spaced along a chromosome such that they home independently but are unlikely to segregate via recombination (Figure 2(c) and S5). This means that successful homing events simply add to any existing constructs on the target chromosome. The exact tolerable spacing between target sites is likely species‐specific, but they must be far enough apart to possess unique homology arms that allow independent homing for each construct yet not so far as to compromise target site linkage. In practice, this likely means a spacing of a few kilobases would be sufficient. This concept is similar to releasing separate drives except that the constructs may become linked over time. Therefore, HDR could copy the entire homologous sequence between two simultaneous DSBs (including any transgenes, resistance and intact target sites in this region). However, less desirable is the possibility for NHEJ repair to delete the entire sequence between two simultaneous DSBs.

#### 2.1.4 Overwriting strategy

In this strategy, each construct will be targeted to the site encoded by its own gRNA. Successful homing reactions will restore the target sites for all other multiplex constructs (see Figure 2(d) and S6). In theory, this could be achieved in several ways, for example through homology arms designed such that homing removes all endogenous target sites but designing each multiplex construct such that it contains the target sites for each of the other constructs. Alternatively, the locus of each construct could be offset from one another such that the homology arms of each construct represent the homologous wild‐type sequence of each other construct. In both cases, successful homing by any construct will delete any existing constructs and/or resistant alleles carried on the homologous chromosome so long as all target sites are located close enough to allow constructs to target and “overwrite” each other. In practice, this overwriting strategy will likely be difficult to engineer given currently available tools but is included here in case future work or tools are able to make this design feasible.

#### 2.1.5 Blocking strategy

This is our preferred strategy based on subsequent results. Here, a successful homing event by one construct removes or disrupts the target sites of other segregating constructs (see Figure 2(e) and S7). As in the overwriting strategy, this requires target sites to be located close enough together that they can all be deleted/mutated in a single successful homing event. As for the overwriting strategy, the tolerable spacing of these constructs is currently unclear. This strategy prevents the possibility of multiple simultaneous DSBs on the same chromosome since the presence of one construct “blocks” the homing action of others, preventing them from becoming linked.

### 2.2 Comparison of neutral‐locus multiplexing approaches

As an initial comparison between the various neutral‐locus multiplexing approaches considered, we perform numerical simulation of each approach when considering one, two, three or four gRNAs. Note that in all cases, the one gRNA case reduces to a simple CRISPR “global” gene drive system and is given to assess the scale of improvement offered by any strategy. Specifically, Figure 2 presents a representative sample of ten numerical simulations for each approach in a population size of 1,000 individuals. Consideration of a biologically realistic population size would present a large computational load; however, our work allows for an initial comparison between approaches. Also, since an increasing population size has been shown to produce an increased probability of failure (Marshall et al., 2017), any approach that is unable to control a population of 1,000 individuals would not be suitable for consideration in any biologically realistic population. Note that these results are produced using a single parameter set outlined in Materials and Methods. Alterations to this parameter set would likely affect the performance of the approaches considered; however, the results here should provide a good indication of the performance of each approach with respect to one another.

Figure 2(a) shows that the addition of extra gRNAs into a multiplexed CRISPR gene drive does not substantially improve its efficacy when targeting a neutral locus. This is likely due to a high cutting rate (85%) giving a large probability of multiple DSBs being made within the same chromosome, thus giving the possibility of NHEJ repair deleting the intervening sequence, as seen empirically in Brinkman et al. (2018), Kistler et al. (2015), Oberhofer et al. (2018). This results in resistance for each target site located between the outermost DSBs, thus allowing multiple resistant alleles to be formed in an individual within one generation. For comparison with other strategies, some summary statistics are shown in Figure S2.

Alternatively, if the expression of each gRNA and subsequent repair of its DSB were sequential, then the possibility of multiple simultaneous DSBs on the same chromosome would be eliminated. As seen in Figure 2(a), this results in a much more effective system. There are, however, major technical hurdles that would need to be overcome for such a system to be feasible. In particular, the ability to fine‐tune the expression of each gRNA to the point that it could be expressed, fully degraded and any DSBs repaired prior to the next gRNA becoming active would be extremely challenging given current knowledge and available tools.

For the overwriting and blocking approaches, each successful homing event results in the homologous chromosome carrying only one construct meaning multiple simultaneous DSBs on the same chromosome (and deletion of the intervening sequence) are eliminated. However, to avoid drive constructs targeting each other in the release generation (potentially altering the introduction frequencies of some constructs) we consider the introduction of *n* distinct pools of individuals each heterozygous for only one of the *n* constructs, while maintaining an overall release ratio of 0.05. We consider a similar release strategy for the additive approach to avoid the initial linkage of individual constructs and also for the separate strategy to enable a fair comparison to be made with the other approaches.

Results for the release of separate drives are shown in Figure 2(b). These show a modest improvement in the efficacy of this strategy from the addition of further constructs. We also find an improvement over classical multiplexing in both the time spent at high frequency (>0.9 transgenic) and until it falls below a transgene carrier frequency of 0.1 (Figure S2). While this strategy represents an improvement over classical multiplexing when targeting a neutral locus, the degree of improvement observed is unlikely to be sufficient to enable the control of realistically sized wild populations.

Sample numerical simulations for an additive strategy with both simultaneous and sequential gRNA expression (Figure 2(c)) show that simultaneous gRNA expression results in a decrease in mean performance with the addition of extra target sites. In spite of this, for low gRNA numbers (i.e. 2 or 3) there is a modest probability of the system persisting for over 150 generations—two from ten numerical simulations, whereas the other eight fell to a low frequency more quickly on average than a single‐target site system. A four‐gRNA approach showed no examples in which the system persisted throughout the numerical simulation window, and the persistence was also less than that of a single‐target site system (confirmed by summary statistics in Figure S2). For sequential gRNA expression, we see a much larger probability of the system persisting, and even where the system does fail, the average persistence is an improvement over a single‐target site system. Summary statistics in Figure S2 appear to show that this approach performs less favourably than classical multiplexing, with this deficit increasing as the number of gRNAs is increased. This is likely due to two main factors. Firstly, multiplicative fitness costs between transgenic constructs produce very large fitness effects on individuals carrying multiple transgenic constructs, whereas classical multiplexing allows only the fitness effect of a single construct. Secondly, as mentioned above, constructs may become linked over time allowing the possibility of multiple simultaneous DSBs and the deletion of multiple target sequences within a single generation. Here, the results of a release of a single pool of individuals heterozygous at all target sites (Figure S3) appear similar to those in Figure 2(c).

Both the overwriting and blocking approaches (Figure 2(d) and (e), respectively) demonstrate a substantial improvement over the simultaneous‐cutting classical multiplexing, separate and additive strategies. Most simulated introductions show a sustained increase in frequency such that every individual carries at least one transgenic construct until at least 150 generations after the time of release. However, for both approaches, some simulated introductions fail to persist. To more accurately assess these failure rates, 10,000 numerical simulations were conducted for two‐, three‐ and four‐target site cases with a population size of 1,000 individuals (summarized in tables within Figure 2(d) and (e)). This shows that, for both approaches, the failure rate is reduced by one or more orders of magnitude for each additional site targeted—under the parameter set considered here (see Materials and Methods). While failure rates appear very similar between blocking and overwriting approaches, a comparison of failed introductions (Figure S4) shows that, on average, the blocking strategy persists at high frequency for slightly longer than the overwriting strategy.

### 2.3 Effects of fitness costs and end‐joining rate

Previous literature has demonstrated the importance of a range of parameters on the outcome of a gene drive release (Alphey & Bonsall, 2014; Deredec et al., 2008; Noble et al., 2017, 2018). Here, we extend the above investigation to explore the effects of fitness costs and the ratio of NHEJ to HDR repair of DSBs. From this point forward, we restrict our attention to the blocking strategy only since it is feasible with currently available tools, outperforms the other strategies studied here and should prove simpler to engineer.

In order to investigate the effects of fitness costs and NHEJ repair probabilities, we first construct a discretized parameter grid ranging between homozygote fitness cost parameters in the range of 0–0.5 and NHEJ repair probabilities between 0 and 0.2. Note that an assumption of additive fitness costs means heterozygotes will incur half of the fitness cost of homozygotes. Also, the probability of repair by HDR will be equal to one minus the probability of NHEJ repair. For each point in this parameter grid, we perform 100 numerical simulations and calculate the mean transgene carrier frequency (i.e. the proportion of the population carrying at least one transgenic construct) 150 generations after the initial introduction.

**FIGURE 3:**
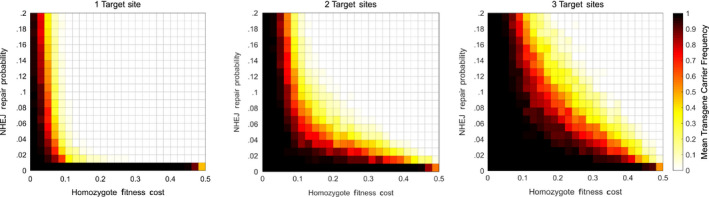
Heat maps showing the mean transgene carrier frequency obtained over 100 numerical simulations of the blocking strategy across a range of fitness cost and NHEJ repair probability parameters. From left to right, these panels represent cases considering one, two and three target sites with the colour bar showing the mean transgene carrier frequency associated with colours shown in the heat maps. Note that the one‐target site system here represents a single‐target CRISPR gene drive with no blocking interaction

In these results (Figure 3), it is clear that the one‐target site CRISPR drive (with no blocking interaction) is able to produce a high transgene carrier frequency that persists until 150 generations after the initial release; however, this is only the case for a small parameter range—with extremely small fitness costs and probabilities of NHEJ repair. For the region producing intermediate mean transgene carrier frequencies, it is likely that in some simulations, the transgene went to fixation, and in others, it was eliminated. Results for the two‐ and three‐target site blocking approaches show an increasing parameter range in which a high mean transgene frequency is observed. In particular, it is clear that increasing the number of target sites allows for much larger fitness costs and greater probabilities of repair by NHEJ while still allowing the respective systems to produce an extremely high mean transgene carrier frequency—representing a very high probability that a given introduction will result in the transgene either reaching fixation or persisting at a high frequency in the population for a large number of generations (at least 150 in the results presented here). As in the one‐target site case, the intermediate mean transgene carrier frequency regions likely represent cases in which some introductions resulted in the transgene going to fixation or persisting at high frequency and some resulted in the elimination of the transgene.

### 2.4 Efficacy in larger populations

**FIGURE 4:**
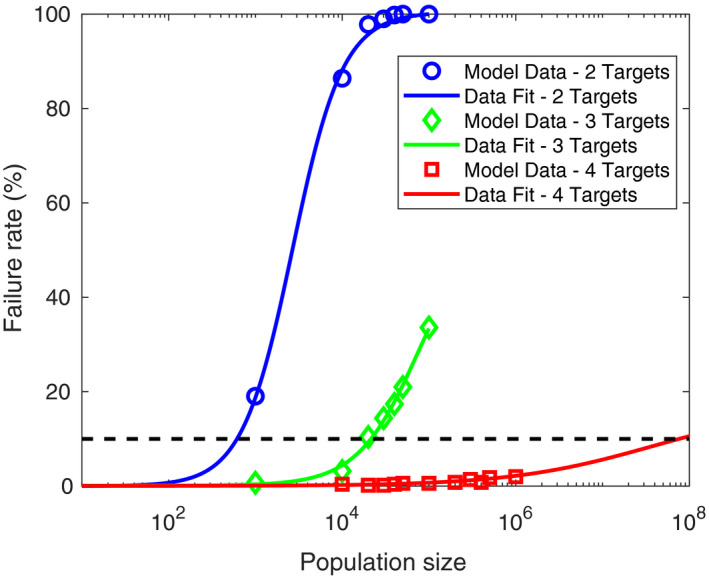
Failure rates for two‐target (blue), three‐target (green) and four‐target (red) site blocking approaches display a sigmoidal dependence on the population size. Symbols display the failure rates obtained from 500 numerical simulations of each strategy for a given population size, whereas lines display a sigmoidal curve fit to each data set. The black dashed line represents a threshold failure rate of 10%

The simulations above used a relatively small population size (1,000 individuals) to allow rapid investigation and comparison between strategies. Increasing the population size presents a greater challenge for CRISPR gene drives as it increases opportunities for fully resistant individuals to emerge, likely raising the failure rate (Marshall et al., 2017). Figure 4 shows failure rate estimates obtained using 500 numerical simulations for a range of population sizes while all other parameters remain equal.

These results suggest that failure rates display a sigmoidal relationship with an increasing population size with rates asymptotically approaching 100% at higher population sizes. Taking a lead from Marshall et al. (2017), we consider conditions giving a 90% chance of a successful introduction (i.e. a failure rate of 10%). Thus, our results suggest that, for the parameter set considered here, a two‐ or three‐target site blocking approach could be used to control a population of approximately 600 or 20,000 individuals, respectively. For the four‐target site blocking approach, the population size leading to a 90% rate of successful introduction was larger than practical for the purposes of numerical computation. We extrapolate the numerical simulation results by fitting a sigmoidal function using a simple least‐squares regression (see Materials and Methods for further details). This suggests that a four‐target site blocking strategy, under the parameter set considered here, would be capable of effectively controlling a population of approximately 75 million individuals. Assuming we continue to see similar degrees of improvement from the addition of further target sites (and gRNAs), we would expect this strategy would be capable of transforming populations over extremely large geographic scales with plausible numbers of gRNAs. However, there are of course numerous environmental, ecological, behavioural and genetic factors beyond the scope of this study that may impact upon the maximum population size that can be controlled by a given multiplexing strategy.

## 3 DISCUSSION

Here, we confirm that multiplexing at the individual level (i.e. incorporation of multiple gRNAs into a single construct) is unlikely to be effective for a CRISPR gene drive targeting a neutral locus. This is due in part to the potential for multiple simultaneous DSBs on the same chromosome to be repaired via an NHEJ deletion of the entire sequence between DSBs, creating resistance at all internal target sites. We found that sequential expression and action of gRNAs significantly improve a classic multiplexing strategy; however, such a system is likely to be prohibitively difficult to engineer given currently available tools. As such, we proposed a number of designs where it is possible for multiplexing to instead take place at the population level (i.e. incorporation of each multiplexing gRNA into an independently segregating construct). An initial comparison of these approaches was conducted using numerical simulation in a small population of 1,000 individuals. This was sufficient to give an initial indication since failure rates have previously been shown to increase with population size (Marshall et al., 2017). Therefore, any approach that is ineffective in the small population should not be taken forward for further consideration in larger populations. Here, a separate strategy in which multiple independently segregating CRISPR gene drives are introduced gave a modest improvement over classical multiplexing. However, this improvement only extends as far as a slight increase in the number of generations for which the system persists at high frequency. An additive strategy showed a decrease in performance compared with the classic multiplexing strategy. This was likely due to the ability of constructs to become linked, enabling the system to produce multiple simultaneous DSBs and repair these via NHEJ deleting the entire intervening sequence. The future possibility of sequentially expressed gRNAs would significantly improve the performance of this strategy by removing the ability to make multiple simultaneous DSBs. Blocking and overwriting strategies—where construct design forces multiplexing to occur exclusively at the population level—gave large improvements over classic multiplexing, separate CRISPR drives and the additive strategy. These approaches produced similar failure rates in a modestly sized population. Since the blocking strategy appears simpler to engineer, we then investigated the performance of this strategy under alternate parameter scenarios and in larger populations. Perhaps unsurprisingly we found that increasing population size leads to increased failure rates for a system with a given number of target sites. The addition of further gRNA targets into a blocking strategy was also shown to substantially lower the failure rate for a given population size.

Target wild population sizes, acceptable persistence times and failure rates are likely to vary considerably between potential applications and target species. However, we are able to make broad comparisons with previous studies of CRISPR‐based gene drives targeting multiple sites. In particular, for neutral‐locus suppression systems in invasive vertebrates (Prowse et al., 2017), a dominant female‐to‐male sex‐reversal gene drive multiplexed with up to five gRNAs was unable to cause the extinction of a mouse population of 50,000 individuals, primarily due to resistance formation. While acknowledging the differences between our two modelling approaches, it is of interest that our four‐gRNA blocking strategy modelled in the same population size and with a common NHEJ rate was able to drive the frequency of transgenic individuals to over 90% for at least 150 generations (and, in the vast majority of these iterations, to elimination of the wild‐type allele) in >99% of simulations. Assessing the shorter term dynamics, which would be more relevant to a population suppression strategy, the same blocking model run with two gRNAs was able to reduce the number of nontransgenic individuals to four or less at generation 16 in >99% of iterations. If such constructs were spreading dominant sex‐reversal genes, this would be equivalent to two or fewer remaining females, by which point demographic effects would likely have led to population collapse. In contrast, Marshall et al. (2017) modelled a classical multiplexing system targeting a gene essential for female fertility (i.e. a non‐neutral locus). They found that for a 90% chance of success, multiplex numbers of two and three were sufficient to eradicate populations of ~10,000 and ~1,000,000, respectively. These appear significantly better than the results shown here for two‐ and three‐target site blocking systems (~600 and ~20,000 individuals, respectively). However, moving to a four‐target site blocking strategy would suggest that we see here a larger increase in performance from the addition of further gRNAs than was the case in Marshall et al. (2017). This, combined with the result that classical multiplexing is unlikely to be successful for any realistic multiplex number when targeting a neutral locus, suggests the strategies proposed here may be worth pursuing in this context.

In addition to this pure resistance management benefit, population‐level multiplexing as described here provides a number of potential technical advantages over classical multiplexing designs that operate at the individual level. For example, it has been observed in experiments testing classically multiplexed CRISPR constructs in *Drosophila* that resistance management effects of additional gRNAs are not additive; that is, rates of HDR did not increase to levels predicted if each DSB was being repaired independently—possibly due to Cas9 protein becoming a limiting factor when shared among multiple gRNAs (Champer et al., 2018). Similarly, it has been postulated that where multiple gRNAs are expressed simultaneously there may be a tendency for particular gRNAs to be preferentially loaded into Cas9, in effect reducing cutting rates and therefore resistance management benefits of other gRNAs within the multiplex cassette. The same authors identified a substantial variation in homing rates using a classically multiplexed CRISPR drive, which they propose may be explained by the interference of multiple Cas9/gRNA complexes bound to the target site with one or more aspect of the HDR repair pathway (Oberhofer et al., 2018). All of these issues are a result of expressing more than one gRNA simultaneously within the same cell, which does not happen in our preferred blocking strategy.

As with any mathematical modelling study, this work is based on a number of simplifying assumptions. In terms of the experimental design, we mimic a laboratory cage‐based experimental strategy similar to that in Hammond et al. (2016, 2017, 2018), Harvey‐Samuel, Ant, Gong, Morrison, and Alphey (2014), which allows us to exclude a range of environmental phenomena (e.g. overlapping generations, fluctuating population size, density dependence, predation and climate/seasonality). The experimental approach on which this work is based was developed to assess the performance of genetically modified insects. While we anticipate that the general results shown here should hold for noninsect species, future work could seek to adjust this model to provide species‐specific predictions for alternative genetic control targets such as mice, rats, fish and other vertebrates (Backus & Gross, 2016; Dearden et al., 2018; Gould, 2008; Prowse et al., 2017; Thresher et al., 2014). One factor potentially requiring adaptation is that each individual is assumed to mate only once; however, if there is no difference between the sexual competition of transgenic and wild‐types, then we do not anticipate that this will drastically alter these results.

There are also some simplifying assumptions applied to elements of the genetic systems themselves. Firstly, we assumed that in individuals heterozygous for two different constructs where homing is still possible (i.e. the overwriting strategy), only one is able to cut the homologous chromosome (i.e. one construct is randomly selected and assumed to act first, thereby disrupting the other construct). For the classic multiplexing, separate and additive strategies, we also assumed that when attempting to induce multiple DSBs on a single chromosome, the action of a Cas9/gRNA complex at one target site will not negatively impact on the efficacy of similar Cas9/gRNA complexes at other target sites. Furthermore, we exclude the possibility of DSBs being repaired through partial/incomplete homing events (Oberhofer et al., 2018). Future incarnations of this model could seek to provide a more detailed description of the precise genetic action of each transgenic construct in this regard. Finally, as for other work in this field to date, we have not considered the possibility of maternal deposition of gRNA‐loaded Cas9 cutting target site(s) on the paternally inherited chromosome (Champer et al., 2017). We expect that careful choice of germline promoters and/or nuclease stability will reduce this risk in strains eventually selected for potential use (Galizi et al., 2014). Additionally, the strategies described here are likely to be equally effective at managing resistance if male‐specific germline promoters are selected: a design which will likely reduce this risk to near zero (Champer et al., 2018). Another potential issue here is that a truly neutral locus may be subject to a large degree of standing genetic polymorphism or perhaps even absent in some individuals. Similar effects may also result from large deletions created during the repair of DSBs following CRISPR/Cas9 nuclease action. Such large deletions were found to occur in mammalian tissue culture cells (Adikusuma et al., 2018; Kosicki, Tomberg, & Bradley, 2018), but not observed in mosquito gene drive experiments (Hammond et al., 2017, 2018); the rate and relevance are hard to estimate at present and may vary between species and loci.

Here, we have presented a range of novel strategies that may be used to improve the efficacy of a CRISPR‐based gene drive targeting a neutral locus. The two most potent of these designs—overwriting and blocking—benefit from the fact that their multiplexing action is forced to function exclusively at the population rather than individual level, precluding the multiple DSBs that reduce the efficiency of classical strategies. Our proposed designs vary in engineering difficulty; however, the most thoroughly studied example (the blocking strategy) should be straightforward to develop. While we have investigated these strategies using a stochastic mathematical modelling framework, further work is required to determine how such systems will behave for specific target species and in more complex environments.

## 4 MATERIALS AND METHODS

### 4.1 Simulated experimental set‐up

Results presented here mimic cage experiments such as those in Hammond et al. (2016, 2017, 2018), Harvey‐Samuel et al. (2014) in a stochastic mathematical model developed and run in MATLAB (version R2016a; The MathWorks, Inc.). A fixed population of 1,000 individuals with 1:1 male‐to‐female ratio was considered (unless otherwise stated). Initially, transgenic individuals were added to a wild‐type population at a predetermined ratio. Individuals were then paired for mating, and offspring produced with genotypes dependent on those of the parents. For each individual, effects of each gene drive and their associated fitness costs were simulated, in the order from Unckless et al. (2015), Unckless et al. (2017). From resulting offspring, 1,000 individuals (500 male and 500 female) were selected to seed the next generation. This was repeated for 150 generations with the transgenic frequency recorded in each. More detailed information is given in Supplementary Information.

### 4.2 Parameter values

We use a reference set of parameters for all numerical simulations unless stated otherwise. The release ratio for all approaches studied here must be equal to allow a fair comparison. Thus, a release ratio of 0.05 was chosen (giving 0.025, 0.0167 and 0.0125 per construct for 2, 3 and 4 target sites, respectively). This was chosen such that all constructs avoid stochastic loss during early generations following release. Such release ratios may exceed the extremely small values used in deterministic studies (Noble et al., 2017; Unckless et al., 2015), but they are still very small compared with those required for many other approaches and approximately half of that considered by Noble et al. (2018). The probability of repair via NHEJ was chosen to be 0.02 to mimic other multiplexing studies (Prowse et al., 2017). For all numerical simulations, we consider a cleavage rate of 0.85. Alongside the rate of NHEJ, this produced a homing rate in this study that represents a middle ground between the modest rates from *Drosophila melanogaster* (Oberhofer et al., 2018) and the very high rates seen in *Anopheles gambiae* mosquitoes (Hammond et al., 2018). As is common in mathematical modelling of gene drives, we consider fitness costs to be additive (i.e. dominance of fitness cost = 0.5). Finally, since we consider the targeting of neutral loci, we would not anticipate extremely large fitness costs. However, we consider a fitness cost of 0.15 so that gene drive carriers have a fairly significant deficit relative to wild‐type individuals.

### 4.3 Release methodologies simulated

Two different strategies were considered for the introduction of transgenic individuals. For the classic multiplexing and separate strategies, individuals heterozygous at all target sites were introduced into a wild‐type population. For additive, overwriting and blocking strategies, the release ratio was divided between the *n* target sites (rounded down to the nearest whole individual) and *n* separate pools of individuals heterozygous at a single target site introduced. For simplicity, we assume the release of both males and females in a 1:1 ratio.

### 4.4 Presentation of results

Results were presented as a proportion of individuals carrying one or more copies of any transgene to aid with future experimental validation. This is because experiments usually score presence/absence of transgenes via fluorescent markers, but to distinguish between heterozygote and homozygote individuals is more difficult.

### 4.5 Calculation of failure rates

A simulated introduction of transgenic individuals was deemed to have failed if the frequency of individuals carrying one or more transgenic construct was less than 0.9 after 150 generations. This extremely stringent measure was used such that results represent a worst‐case scenario.

### 4.6 Data fit to numerical simulation results

Sigmoidal functions of the form.

$$f\left(x\right)=A+\left(\frac{B-A}{1+{\left(\frac{C}{x}\right)}^{D}}\right),$$

where *x* represents the population size, were fitted to numerical simulation data in the form of an aggregated failure percentage from 500 simulations at each population size considered. This data fit was via a simple least‐squares regression performed using the Microsoft Excel Solver add‐in subject to the constraint that 0 ≤ *f*(*x*) ≤ 100 for all *x*.

## CONFLICT OF INTEREST

The authors have submitted a provisional patent application for this technology.

## Supporting information

Supplementary Material

## Data Availability

MATLAB code for the models will be made available without restriction upon request to the authors.
