# Supplementary material for: Population‐level multiplexing: A promising strategy to manage the evolution of resistance against gene drives targeting a neutral locus
Source: Evol Appl. 2020 Mar 25;13(8):1939–48. doi: 10.1111/eva.12945 (PMC7463328; doi:10.1111/eva.12945)
Supplement: Supplementary file 1 — Supplementary Material [file EVA-13-1939-s001.pdf]

Supplementary Information for:
Population-level multiplexing effectively manages the evolution of resistance against gene drives targeting a neutral locus

As discussed in the main text, we mimic a laboratory cage experimental setup similar to those in [1, 2, 3, 4]. This type of experimental setup is numerically simulated using a stochastic mathematical model coded in MATLAB (version R2016a, The MathWorks Inc., Natick, MA). Here we discuss aspects of this model formulation in chronological order to give an insight into its function.

Initialisation

The MATLAB script used within this study begins with the definition of all relevant parameters (as listed in Materials and Methods in the main text). Following this, the script initialises a population with two matrices of dimensions $500{\times}2n$ (i.e. the number of male or female individuals by two times the number of target sites). This matrix is then populated according to the alleles present at each of those target sites with ones, twos and threes denoting wild-type, transgene and resistant alleles, respectively. As such, we begin with $500{\times}2n$ matrices containing all ones to represent fully wild-type laboratory cage population. The matrices are then appended with a number of transgenic individuals according to the defined release ratio - chosen to be r=0.05 throughout this study. These transgenic individuals are added in one of two ways depending on which approach is used to target multiple sites. For classic multiplexing and the separate approach, individuals heterozygous at all target sites are added to the population at release ratio r - shown in the left panel below. However, for the additive, overwriting and blocking approaches, individuals are released in n pools each heterozygous at one target site at a release ratio of r/n (rounded down to the nearest whole individual) - as shown in the right panel below. Note that in both release scenarios we consider similar additions of individuals to both the male and female matrices. Finally, we provide MATLAB with a random number generator seed that is based on the current time in order to avoid repeated use of the same random number set.

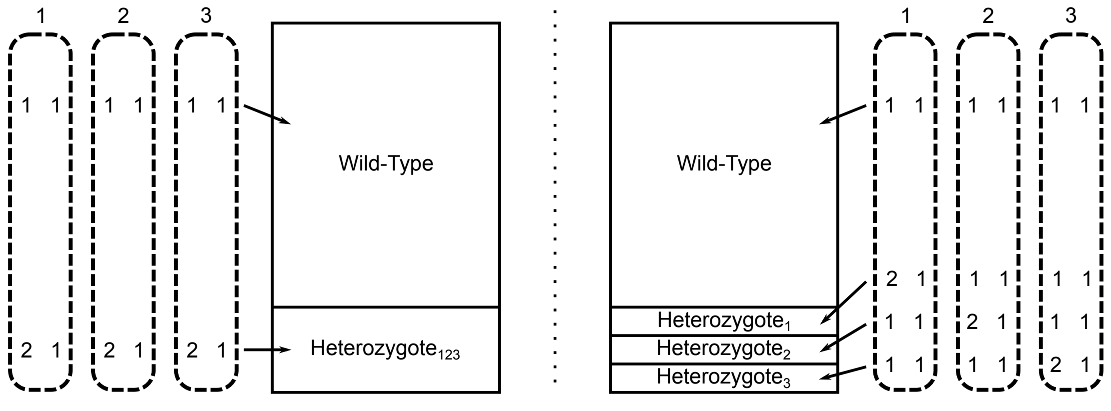

Mating and offspring

The first step within each simulated generation is to assign individuals into mating pairs. This is done by matching each males with a randomly selected female - with each female assigned to a single male only. From each of these mating pairs we then create 15 offspring individuals each of which is randomly assigned a genotype based on the list of possible outcomes from the parental genotypes. The 15 offspring individuals from each mating pair are then grouped and stored in a single matrix with a similar structure to those pictured above so as to create a pool of individuals from which the next generation will be selected.

Gene drive effects

The next stage of this simulation process is the simulation of homing in each individual. This study considers a range of approaches creating their own unique set of rules for how this process should occur. In the first instance each of the approaches considered decide which target sites undergo double strand breaks (DSBs). This process begins by examining the alleles present at a particular target site on each chromosome in an individual to determine whether all of the required components for a DSB to be made are present. Specifically, one chromosome carries the transgenic construct and a wild-type allele for that target site on the homologous chromosome (i.e. a (2,1) or (1,2) pair for a given target site in the offspring matrix) then a DSB could be made. In this event, a number in the range zero to one (inclusive) is randomly selected and compared to the parameter determining the probability of an endonuclease cutting. If this random number is greater than or equal to the cutting probability parameter then a potential cut is recorded or else no cutting may occur. This is repeated for each target site and each direction of cutting with results stored in two arrays containing lists of target sites with potential DSBs on each chromosome.

Classical multiplexing

The first approach considered in the main text is the classical multiplexing approach in which the multiple gRNAs are carried on a single transgenic construct. The most common consideration for this type of approach is that each gRNA should be expressed simultaneously, however it is possible to conceive of an alternative in which gRNA expression is tightly regulated such that each is expressed sequentially. The differences between these are discussed in the sections below.

Classical multiplexing with simultaneous gRNA expression

Here the sizes of the arrays containing potential DSB locations on each chromosome are checked. In the event that both arrays contain one or more elements (indicating potential DSB sites on each chromosome) then a random number (1 or 2) is selected to determine which chromosome actually undergoes DSBs. In

simple terms, it is assumed that cutting and repair may only be in a single direction within one individual in each generation. Once a direction of cutting has been chosen, the number of DSB cut sites is checked.

For cases with a single DSB site, a random number is selected and compared to the parameter determining the probability of repair by NHEJ at a single DSB. If this random number is less than or equal to the NHEJ repair parameter then the DSB is assumed to undergo repair by NHEJ (i.e. the entry in the offspring matrix is converted to a 3 (resistant) for that target site). Otherwise repair is assumed to be via HDR copying across the entire multiplexed transgenic construct (i.e. entries for all target sites on the repaired chromosome are converted into the number 2 (transgenic)).

Alternatively, in examples with multiple DSB locations we select a random number and compare this to the parameter determining the probability of NHEJ repair between multiple DSBs. If this random number is less than or equal to the multiple DSB NHEJ repair parameter then repair is assumed to be via NHEJ, thus deleting the entire region in between the outermost DSB sites (i.e. offspring matrix entries for all target sites between and including the outermost DSB locations are converted to the number 3 (resistant)). Otherwise, repair is via HDR copying across the entire multiplexed transgenic construct (i.e. entries for all target sites on the repaired chromosome are converted into the number 2 (transgenic)).

Classical multiplexing with sequential gRNA expression

As in the simultaneous expression case, the arrays containing potential DSB locations for each chromosome are inspected to determine whether there were potential DSB sites on both, one or neither chromosome(s). If both chromosomes contain potential DSB sites then a random number (1 or 2) is chosen to determine which chromosome undergoes a round of cutting and repair. For examples with only a single DSB site, the repair mechanism simulated is precisely as described in the simultaneous gRNA expression case above.

The main difference, however, is in the repair process when multiple DSBs occur. In this sequential gRNA expression strategy, it is assumed that each DSB is made and repaired before the next DSB can occur. As such, the repair mechanism for a chromosome with a single DSB is repeated iteratively for each DSB made. However, in the first instance whereby repair is by HDR, the entire transgenic construct would be copied across (converting all target sites for the relevant chromosome into the number 2 (transgenic)). This would prevent any further DSBs being made and so the first time repair is by HDR, the cut and repair cycle for that chromosome is ended.

Additive approach

Another approach considered in the main text that has the possibility of simultaneous or sequential expression of gRNAs is the additive strategy. In essence this approach may be thought of as splitting a classical multiplexing system such that each gRNA is carried on its own transgenic construct - i.e. multiple

separate drives are considered. The workings of this approach in both the simultaneous and sequential gRNA expression cases are discussed in the sections below.

Additive approach with simultaneous gRNA expression

Here we begin as in the previous approaches detailed above by determining a direction in which cutting occurs. This is achieved via the same mechanism as above (i.e. randomly choosing a number - 1 or 2). In the event of a single DSB, the repair mechanism is as detailed for the classical multiplexing approaches except that a single construct is copied across rather than an entire multiplexed construct (i.e. a single target site in the offspring matrix is converted to a 2 (transgenic)).

For cases whereby multiple DSBs are made, the repair mechanism is selected by comparing a randomly selected number to the parameter determining the probability of NHEJ repair between multiple DSB sites. If the randomly selected number is less than or equal to the multiple DSB NHEJ repair parameter then repair is via the NHEJ deletion of the entire region between the outermost DSB sites (i.e. all target sites between and including the outermost DSB sites are converted to a 3 (resistant)). Otherwise repair is assumed to be via HDR between the outermost DSB sites, resulting in the copying of all constructs between and at the outermost DSB sites (i.e. all target sites between and including the outermost DSB sites are converted to a 2 (transgenic)).

Additive approach with sequential gRNA expression

In the case whereby constructs in an additive approach are expressed sequentially, the DSB and repair mechanism is as described above for repair of single DSBs but repeated for each target site at which a DSB is induced. It is assumed here that the repair of one DSB by HDR or NHEJ do not alter the efficiency of either repair mechanism as subsequent target sites.

Separate approach

For cases in which we consider the introduction of multiple independently segregating CRISPR gene drives each targeting a single sequence, the numerical implementation is extremely similar to that of an additive approach with sequential gRNA expression. The only difference between the numerical implementation of these strategies is that here constructs are allowed to segregate independently, whereas for the sequentially expressing additive strategy all constructs on the same chromosome are inherited together.

Overwriting approach

In this approach with the release strategy in which individuals are released in pools of individuals heterozygous at only one target site, it is not possible for an individual to carry two constructs on the chromosome.

Thus, here the possibility of repair between multiple DSBs need not be considered. As in previous cases, if there are potential DSBs on both chromosomes then we randomly select which undergoes DSBs by randomly selecting a number (1 or 2) to determine the direction of cutting. Once this has been determined either by the presence of only one DSB or the random selection of cutting direction, the repair mechanism is not too dissimilar from those for a single DSB outlined in previous sections. In particular, if a randomly selected number is less than or equal to the parameter for the probability of repair by NHEJ then the DSB is repaired by NHEJ (i.e. the relevant target site is converted to a 3 (resistant)). Otherwise repair is by HDR of the transgenic construct (i.e. conversion of the relevant target site to a 2 (transgenic)). The main difference here is that this approach also re-writes a complete and undisturbed set of all other target sites - replacing any transgenes or resistant alleles held at those sites. This is represented by the conversion of all target site entries for the relevant chromosomes to the number 1 (i.e. wild-type, representing intact target sites).

Blocking approach

Similar to the overwriting approach described above, here releasing pools of individuals heterozygous at just a single target site eliminates the possibility of multiple constructs being carried on a single chromosome. In fact, the simulation of this blocking approach follows the same steps as the overwriting strategy except that during repair be HDR, instead of re-writing a complete set of target sites, this approach deletes all target sites and constructs carried on that chromosome (i.e. all target site entries are converted to the number 3 (i.e. resistant)).

Repair of single and multiple double stranded breaks

Within this study we assume that the rates of homology directed repair (HDR) and non-homologous end-joining (NHEJ) are the same for both examples with a single double stranded break (DSB) and those with multiple. Since there is little experimental literature from which to compare these rates, we justify this assumption conceptually below.

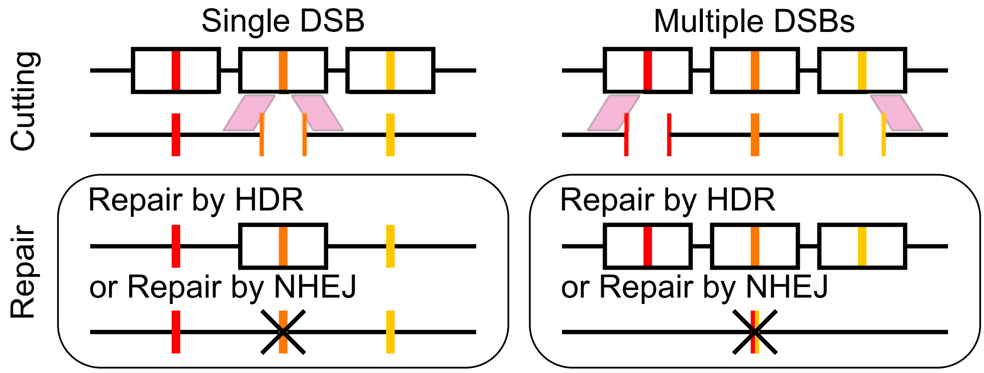

For the simple case of a single DSB the regions of homology (pink areas in the above image) can be used such that the transgenic construct is utilised as a repair template for HDR. An alternative to this is that the broken ends may essentially be ligated together (NHEJ) which often results in small insertion or deletion mutations that create target sites resistant to future cutting.

In the more complex case where multiple DSBs are created, repair may also be via either HDR or NHEJ. Here we assume that any sequence between the multiple DSBs is lost, leaving two repair options. In this case, the regions of homology (pink areas in the above image) should allow the intervening region to be used as a repair template during HDR. Alternatively the two broken ends may simply be ligated together, thus deleting the entire intervening sequence and creating resistance at all target sites between (and including) those where DSBs were induced.

Assuming that the sequence between two DSB sites is lost then there is very little difference between these repair mechanisms other than the size of the region to be repaired. With little to no data available on how the size of region to be copied affects rates of HDR, we assume here that this is equal for repair of single and multiple DSBs and also that it is unaffected by the separation of multiple DSB sites (i.e. the rate of HDR between DSBs at target sites 1 and 2 is equal to that for DSBs between sites 1 and 3). This is an area in which this model could be refined as more experimental data becomes available.

Fitness costs

The effects of fitness costs on the survival of individuals is then simulated. This begins by calculating the relative fitness of each individual according to the equation

$$\Omega = (1 - h_1 s_1)^{\alpha_1} (1 - s_1)^{\beta_1} \dots (1 - h_n s_n)^{\alpha_n} (1 - s_n)^{\beta_n} ,$$

where s_i represents the fitness cost conferred by construct i, h_i denotes the dominance of those fitness costs while $\alpha_i = 1$ and $\beta_i = 0$ if the individual is heterozygous or $\alpha_i = 0$ and $\beta_i = 1$ they are homozygous at target site i. For each individual this Ω value is compared to a randomly selected number $0 \leq R \leq 1$. In cases where $R \leq \Omega$ the individual remains in the pool of offspring whereas if $R > \Omega$ then the individual is removed, representing their failure to survive through to adulthood.

Seeding the next generation

To seed the next generation 500 individuals are randomly selected from the offspring pool to form the male population for the next generation. Similarly, 500 of the remaining individuals in the pool of offspring are selected to create a female population for the next generation. These individuals and their respective

genotype information is then carried forward into a new iteration and the above steps repeated for the desired number of generations - 150 within the context of this study.

Model validation

Within this study we utilise a stochastic mathematical model to predict the efficacy of a range of novel approaches for targeting multiple sites at a neutral locus for population replacement using CRISPR-based gene drive. Since these strategies are novel to this study we are not able to validate these results against deterministic models. However, we are able to validate the general model structure by comparing results obtained from the model of Unckless et al. [5] to those obtained here for a system targeting a single site. As such, we numerically simulate the deterministic model using parameter values detailed in Materials and Methods of the main text and compare the resultant wild-type, transgene and resistant allele frequencies to the average taken over 20 stochastic simulations of our model. This yields the results shown in Fig. S1, confirming that the stochastic model detailed above produces a good agreement with models in the previous literature.

References

[1] Tim Harvey-Samuel, Thomas Ant, Hongfei Gong, Neil I Morrison, and Luke Alphey. Population-level effects of fitness costs associated with repressible female-lethal transgene insertions in two pest insects. *Evolutionary Applications*, 7(5):597–606, 2014.

[2] Andrew Hammond, Roberto Galizi, Kyros Kyrou, Alekos Simoni, Carla Siniscalchi, Dimitris Katsanos, Matthew Gribble, Dean Baker, Eric Marois, Steven Russell, et al. A CRISPR-Cas9 gene drive system targeting female reproduction in the malaria mosquito vector Anopheles gambiae. *Nature Biotechnology*, 34:78–83, 2016.

[3] Andrew M Hammond, Kyros Kyrou, Marco Bruttini, Ace North, Roberto Galizi, Xenia Karlsson, Nace Kranjc, Francesco M Carpi, Romina D'Aurizio, Andrea Crisanti, et al. The creation and selection of mutations resistant to a gene drive over multiple generations in the malaria mosquito. *PLoS Genetics*, 13(10):e1007039, 2017.

[4] Andrew M Hammond, Kyros Kyrou, Matthew Gribble, Xenia Karlsson, Ioanna Morianou, Roberto Galizi, Andrea Beaghton, Andrea Crisanti, and Tony Nolan. Improved CRISPR-based suppression gene drives mitigate resistance and impose a large reproductive load on laboratory-contained mosquito populations. *bioRxiv*, doi: 10.1101/360339, 2018.

[5] Robert L. Unckless, Andrew G. Clark, and Philipp W. Messer. Evolution of resistance against CRISPR/Cas9 gene drive. *Genetics*, 205(2):827–841, 2017.

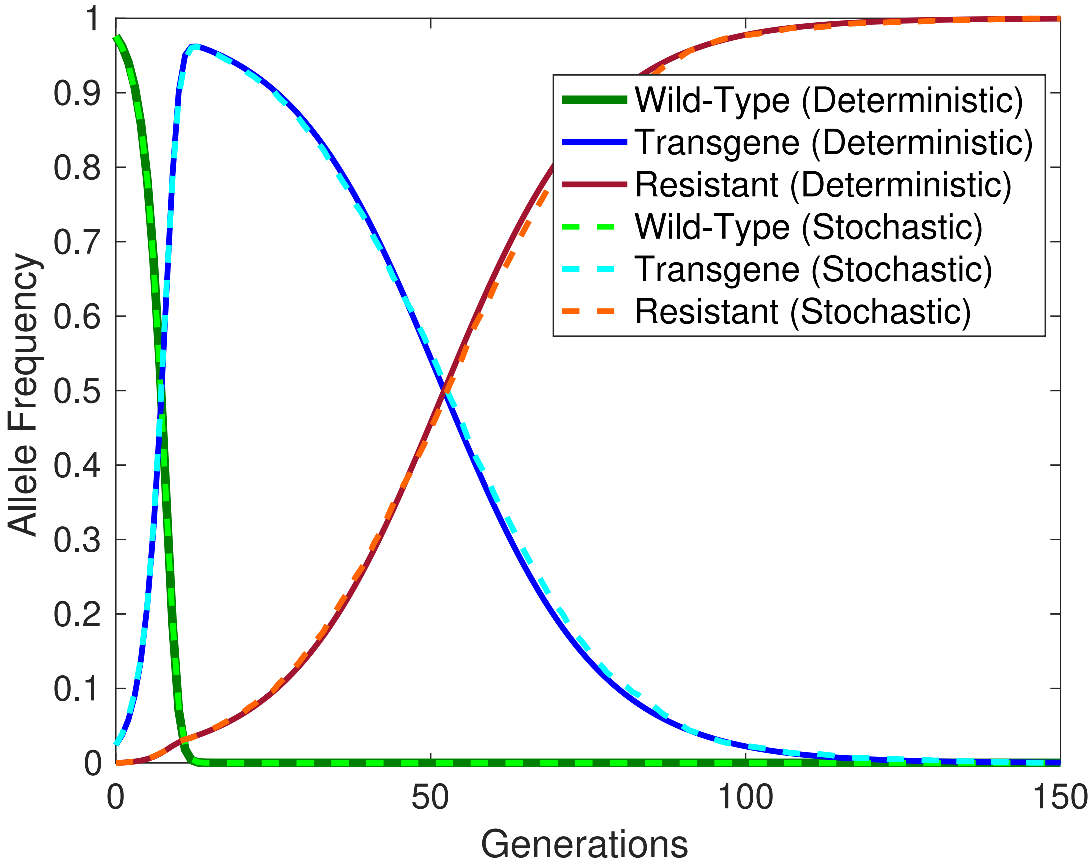

Figure S1: Comparison of deterministic and stochastic modelling results for a CRISPR gene drive system with a single target site. Here solid lines are results from the deterministic model of Unckless et al. [5] whereas dashed lines represent the average of 20 stochastic numerical simulations created using the model developed here. Green, blue and red lines represent the wild-type, transgene and resistant allele frequencies respectively. These results give a good indication that the stochastic model developed within this study provides a good match with previous deterministic models from the literature. Parameter values in this model are: probability of cutting = 0.85, probability of resistance formation by NHEJ given that cutting has occurred = 0.02, transgene homozygote fitness cost = 0.15, dominance of fitness cost = 0.5, fitness cost of resistance = 0 and release ratio = 0.05.

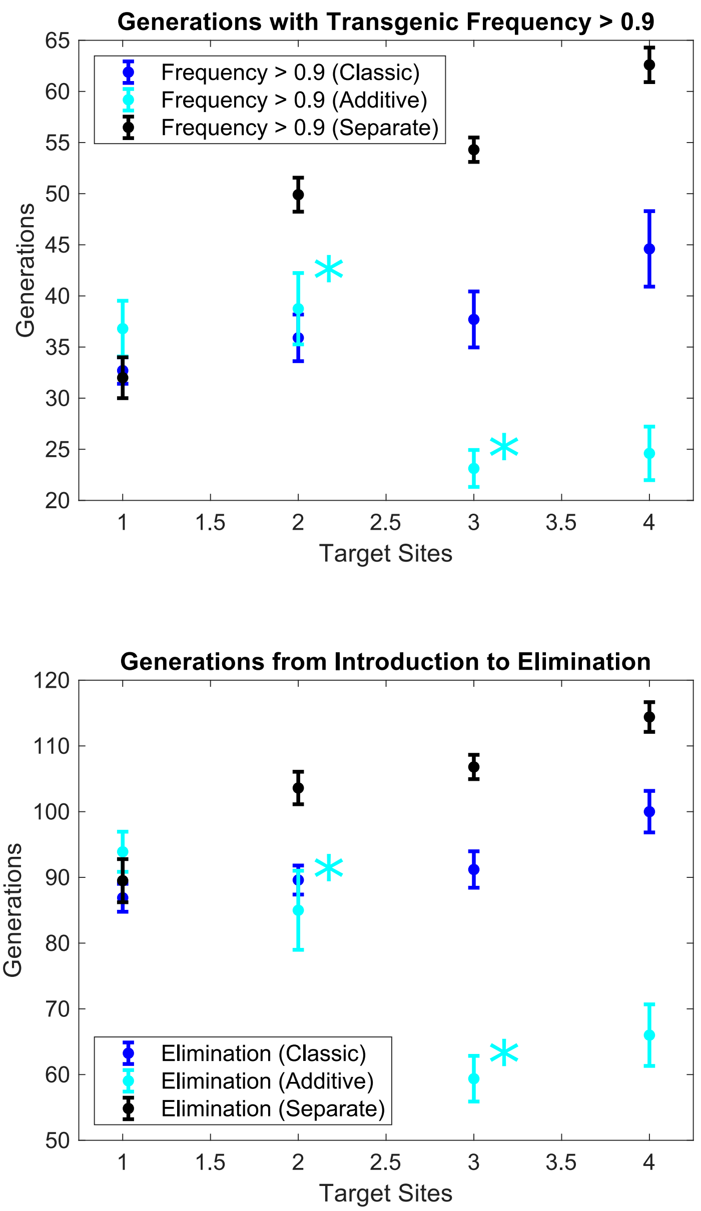

Figure S2: Comparison of summary statistics for classic multiplexing, separate CRISPR drives and the additive strategy proposed in this study. These data points refer to the case of simultaneous gRNA expression only as we do not believe sequential expression to be feasible given currently available tools. Crosses indicate the mean (± standard error) number of generations each strategy maintains a frequency of transgenic individuals above 0.9 (i.e. carriers of one or more transgenic construct) . Circles represent mean (± standard error) time from introduction of transgenic individuals until the elimination of the system (taken as the generation where the frequency of transgenic individuals falls below 0.1). All means and standard errors are calculated from the ten sample numerical simulations presented in main text Figures 3, 4 and 5 except for those labelled with an asterisk which are calculated from the eight numerical simulations that represented a failed introduction (the other two were maintained at a transgene carrier frequency of greater than 0.9 for the duration of the numerical simulation).

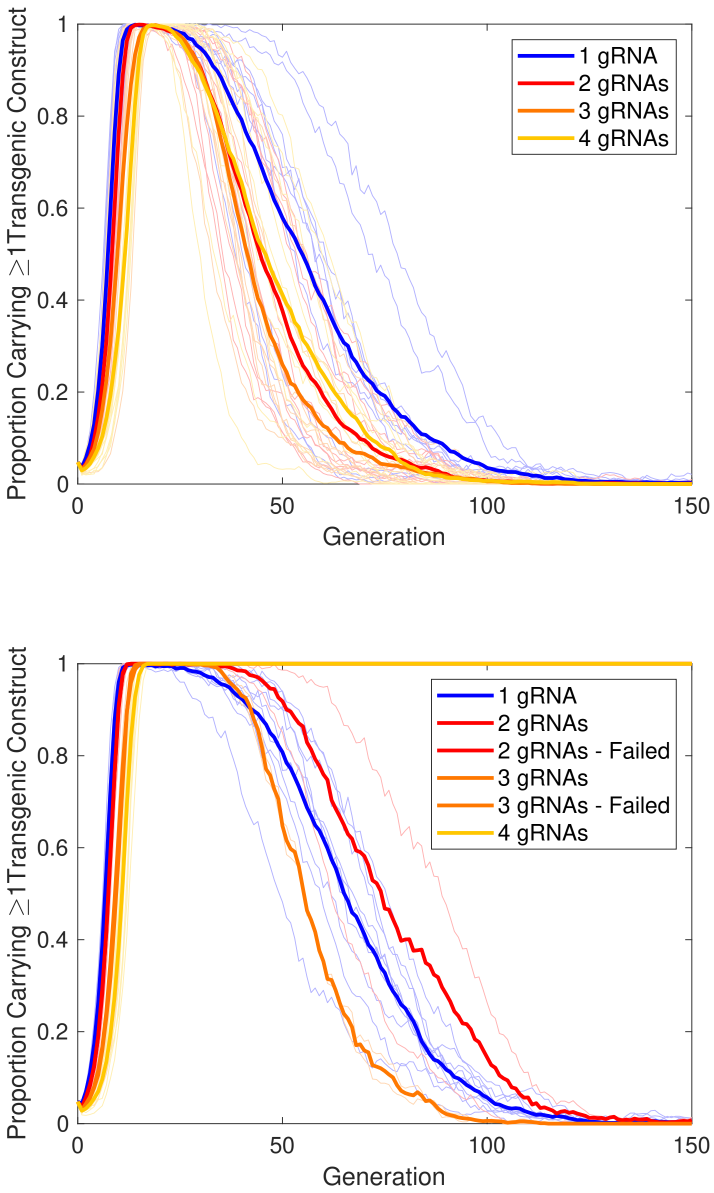

Figure S3: Representative numerical simulations of a CRISPR gene drive targeting n-sites at a neutral locus through an 'additive' strategy with either simultaneous gRNA expression (top) or sequential gRNA expression (bottom). Here introduced individuals are assumed to be heterozygous at each target site simultaneously. Each line represents a single simulation with colors denoting the number of sites targeted - details in the figure legends. Thin lines represent results of an individual numerical simulation whereas thick lines represent the mean of those simulations. Note that for some cases we show two thicker lines - here one represents the successful introductions while the other is for the failed introductions. For comparison, blue lines represent a single target site CRISPR drive targeting a neutral locus. In each case a release ratio of 0.05 is considered (details in Materials and Methods).

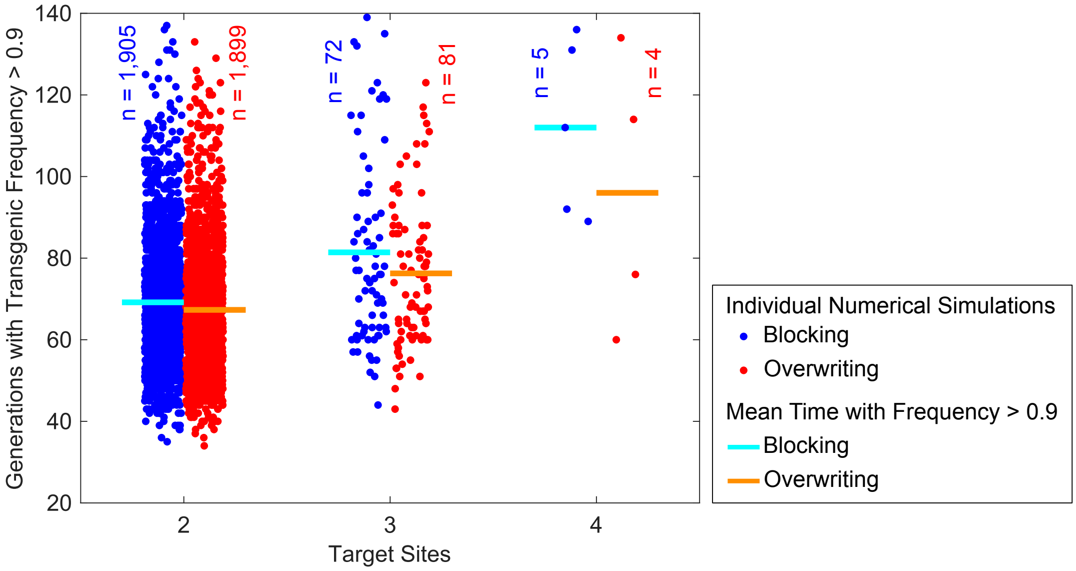

Figure S4: Comparison of some summary statistics for the blocking and overwriting approaches proposed in this study. Here data points represent the number of generations whereby the frequency of transgenic individuals is above 0.9 with the colour denoting either the blocking (blue) or overwriting (red) approaches. Coloured bars indicate the mean of all data points for a given approach and number of target sites. Data points presented here are calculated from the failed introductions listed in main text Figures 4(b) and 5(b), hence the different number of data points for each case.

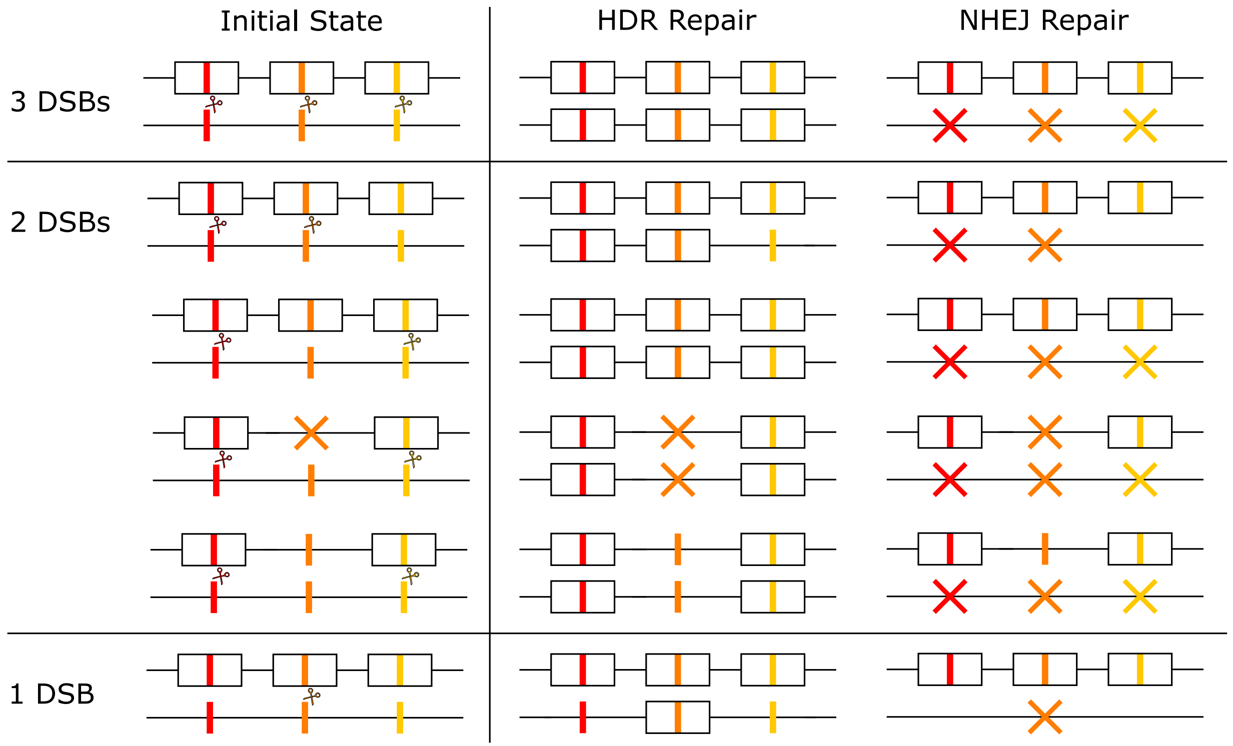

Figure S5: Schematic diagrams of the possible outcomes resulting from the repair of DSBs created by a simultaneously expressing additive multiplexing strategy. Outcomes presented here are for the example of a three target site strategy. Here the left column represents the initial state of the system while the central and right columns show the outcome if repair is by HDR or NHEJ, respectively. In each diagram boxes represent transgenic constructs, unboxed bars denote intact target sequences, crosses show resistant/mutated target sequences and scissor symbols indicate a DSB being made at the associated target sequence.

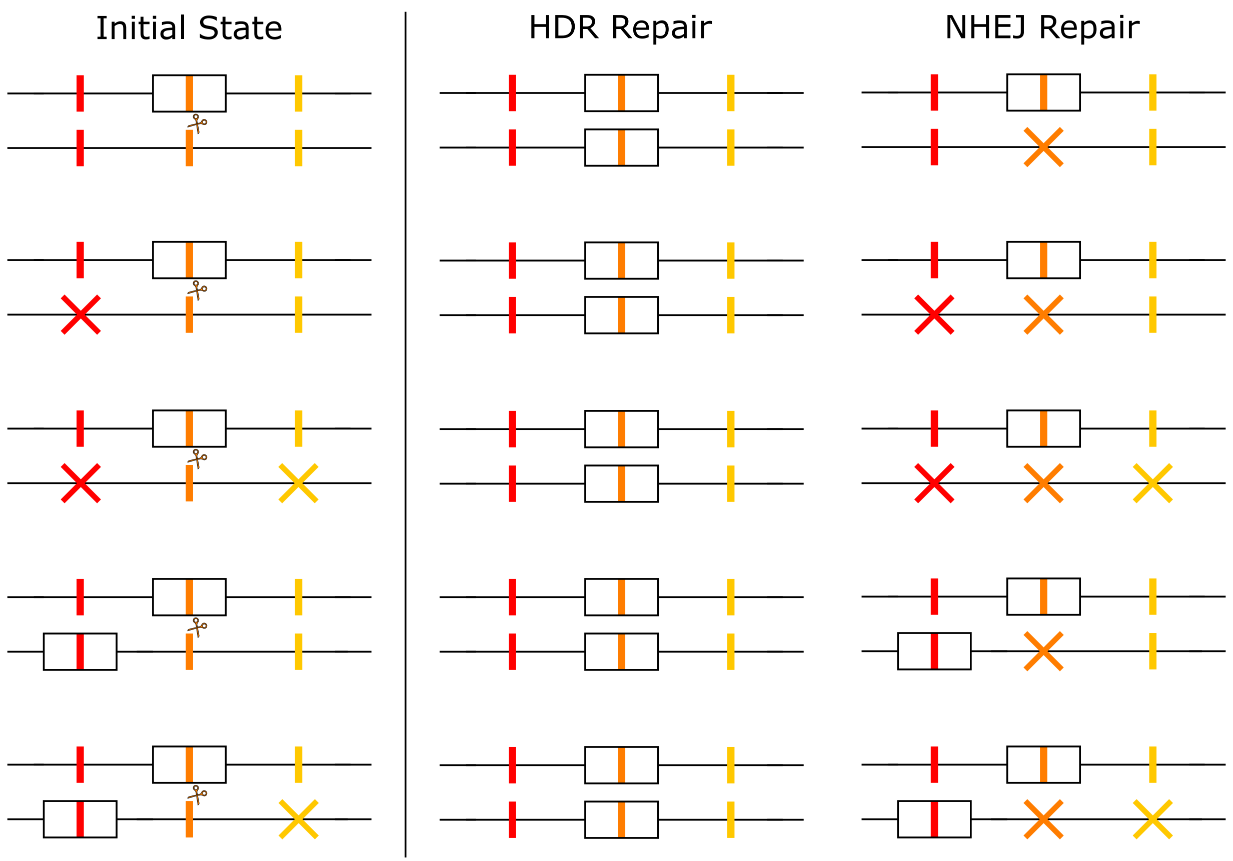

Figure S6: Schematic diagrams of the possible outcomes resulting from the repair of DSBs created by an overwriting multiplexing strategy. Outcomes presented here are for the example of a three target site strategy. Here the left column represents the initial state of the system while the central and right columns show the outcome if repair is by HDR or NHEJ, respectively. In each diagram boxes represent transgenic constructs, unboxed bars denote intact target sequences, crosses show resistant/mutated target sequences and scissor symbols indicate a DSB being made at the associated target sequence.

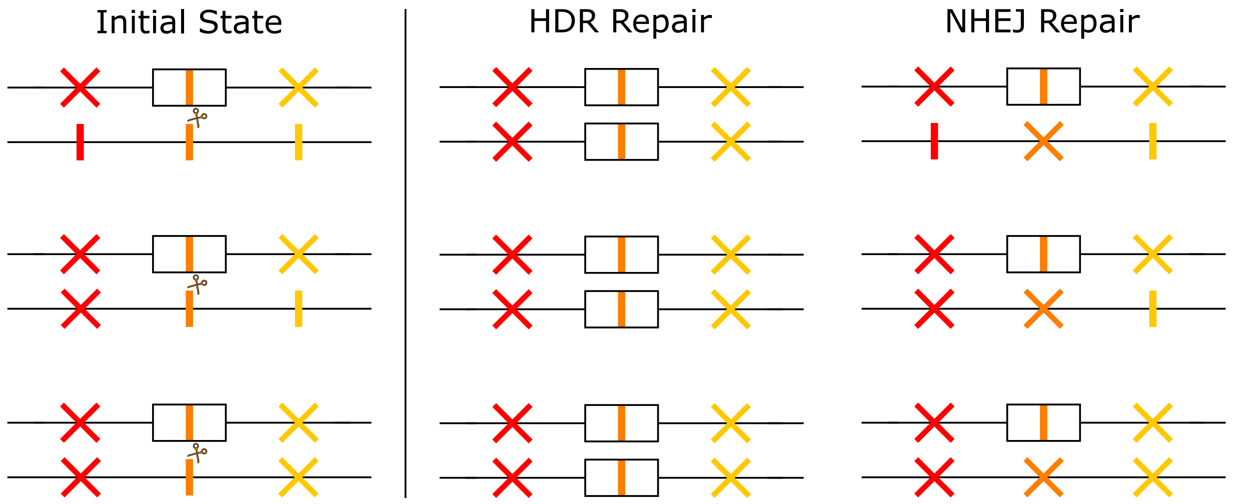

Figure S7: Schematic diagrams of the possible outcomes resulting from the repair of DSBs created by a blocking multiplexing strategy. Outcomes presented here are for the example of a three target site strategy. Here the left column represents the initial state of the system while the central and right columns show the outcome if repair is by HDR or NHEJ, respectively. In each diagram boxes represent transgenic constructs, unboxed bars denote intact target sequences, crosses show resistant/mutated target sequences and scissor symbols indicate a DSB being made at the associated target sequence.
